# Normative volume measurements of the fetal intra-cranial compartments using 3D volume in utero MR imaging

**DOI:** 10.1007/s00330-018-5938-5

**Published:** 2019-01-25

**Authors:** Deborah A. Jarvis, Chloe R. Finney, Paul D. Griffiths

**Affiliations:** Academic Unit of Radiology, University of Sheffield, Floor C Royal Hallamshire Hospital, Sheffield, S10 2JF England

**Keywords:** Fetal development, Magnetic resonance imaging, Prenatal diagnosis, Image processing, Computer assisted

## Abstract

**Purpose:**

To describe the normal linear measurements of the skull (bi-parietal diameter and occipito-frontal diameter) and intracranial volumes (ventricular volume, brain parenchymal volume, extra-axial volume and total intra-cranial volume) in normal fetuses.

**Materials and methods:**

We recruited pregnant women from low-risk pregnancies whose fetuses had normal ultrasound and in utero MR studies. All volunteers had in utero MR imaging on the same 1.5T MR scanner with a protocol consisting of routine and 3D steady-state volume imaging of the fetal brain. Linear measurements of the skull were made using the volume imaging. The 3D volume imaging also was manually segmented to delineate the intracranial compartments described above to determine quantitative values for each.

**Results:**

Two hundred normal fetuses were studied with gestational ages between 18 and 37 weeks. The linear skull measurements made on in utero MR imaging closely correlate with published data from ultrasonography. The intracranial volume data is presented as graphs and as tabular summaries of 3rd, 10th, 50th, 90th and 97th centiles.

**Conclusion:**

It is now possible to measure the volumes of the intracranial compartments in individual fetuses using ultrafast in utero MR techniques.

**Key Points:**

*• There are limitations in using the skull size of the fetus to comment on the state of the fetal brain.*

• *Volumes for the intracranial compartments are presented, based on in utero MR imaging of the fetal brain between 18 and 37 weeks gestational age.*

• *Those normative values can be used to assess fetuses with known or suspected structural brain abnormalities and may assist the differential diagnosis provided by visual assessment of routine iuMR studies.*

**Electronic supplementary material:**

The online version of this article (10.1007/s00330-018-5938-5) contains supplementary material, which is available to authorized users.

## Introduction

The measurement of a range of anatomical structures is an integral part of the ante-natal assessment of the fetus using ultrasonography (USS). This includes assessment of the fetal head size by way of bi-parietal diameter (BPD), occipito-frontal diameter (OFD) and/or head circumference. There are several published growth charts of normative data, to which USS measurements can be compared [1–3]. Head size is also assessed if a fetus is referred for MR imaging due to suspected abnormalities. The published reference values from MR imaging data are limited however, due to a narrow range of gestational ages or small sample sizes [4–7]. USS charts are therefore used as a reference for MR, highlighting the need for further studies.

It is possible to measure some linear brain dimensions (as opposed to skull) on USS but this is not routinely carried out in clinical practice, so skull measurements are frequently used as a surrogate indicator of brain size. That approach is unreliable because growth of the fetal skull is influenced by factors other than growth of the brain per se. For example, it is well established that increased volume and pressure in the cerebral ventricles (fetal hydrocephalus) is usually accompanied by increased skull size because the individual bones of the fetal calvarium are unfused [8]. Linear measurements of the skull may be useful up to a point but it seems intuitively correct that accurate measurement of the intracranial contents will improve the diagnosis of fetal neuro-pathologies. As such, the design and trialing of methods that allow accurate and reproducible measurement of the volumes of intracranial structures is a worthwhile research goal.

There are several ways to divide the intracranial contents anatomically and a frequently used model describes three compartments: the cerebrospinal fluid (CSF) containing ventricular volume (VV), brain parenchymal volume (BPV) and the extra-axial volume (EAV) containing both CSF and vascular structures. These three volumes summated constitute the total intracranial volume (TICV). It is now possible to acquire data in individual fetuses using ultrafast three-dimensional (3D) MR imaging [9–13] that permits the volume of these compartments to be measured after post-processing. This development could be important for accurate diagnosis because different types of fetal neuropathology are expected to affect the compartments in different ways. The first stage in this process is to describe normality.

In this paper, we provide normative MR data for linear head measurements and for the volumes of the intracranial compartments for second and third trimester fetuses between 18 and 37 gestational weeks (gw).

## Materials and methods

### Subjects

The data presented in this paper is derived from a prospective observational study of pregnant women who volunteered to undergo MR imaging of their fetus. In an earlier publication we reported on the BPV (only) from 132 of the cases reported in this manuscript along with a more detailed description of methods [10]. This current work builds on the previous to include the data from 200 fetuses and report the linear skull measurements and the volumes of the intracranial compartments BPV, VV, EAS and TICV.

All of the 200 fetuses in the study were considered to be normal and low risk on the basis of:

- No family history of developmental abnormalities
- No abnormalities (brain or somatic) on ante-natal USS performed after 18gw
- A normal brain on the iuMR imaging study (see below)

### Ethical approval

The pregnant women provided written, informed consent with the approval of the relevant Ethics Board, from two sources; either as funded extension to the MERIDIAN [14] study (Board reference number REC11/YH/0006) or through a second research study sponsored independently by our Institution (Board reference number REC10/H1308/2). Women were not paid for their involvement in the study but travel expenses were provided for them and a companion. The three fetuses with brain abnormalities reported in the online supplemental material were clinical referrals to our Institution and relevant review was sought, and approval obtained, from the Institutional Clinical Effectiveness Unit and Research Department to allow them to be reported.

### MR imaging technique

All iuMR studies were performed at the University of Sheffield’s MR facilities after being screened for contraindications to MR imaging. The iuMR imaging studies were performed between 18-37gw inclusive, the age being calculated from the estimate made on second trimester USS. All studies were performed on a 1.5T whole body scanner (Signa HDx, GE Healthcare) with an 8-channel cardiac coil positioned over the maternal abdomen, with the mother in the supine or supine/oblique position. Maternal sedation was not used and the iuMR studies of the fetal brain were performed within a 30-min table occupancy time. Our standard clinical iuMR imaging protocol (Table 1) was used to acquire 2D images in all three orthogonal planes and 3D data sets were acquired in the axial plane, relative to the fetal brain, using a balanced steady-state imaging sequence (Fast Imaging Employing Steady-state Imaging—FIESTA, GE Healthcare). This short (18–22 s) imaging sequence allows acquisition of the entire fetal brain during maternal suspended respiration [9–12]. All of the iuMR studies were reported by a pediatric neuroradiologist (PDG) with over 18 years’ experience of fetal neuroimaging.

**Table 1:** MR parameters used for fetal imaging (1.5 T GE Healthcare)

	T2 ssFSE	3D FIESTA	DWI	FLAIR	T1	MOVIE
Repetition time	Minimum (2000)	Minimum (4.4)	4000	Minimum (2700)	Minimum (6.2)	4.6
Time to echo	120	Minimum (4.4)	Minimum	122	Minimum (303)	3
Flip angle	–	60	–	–	45	45
Bandwidth (KHz)	37	125	250	41	31	166
Inversion time	–	–	–	2000	–	–
Prep time	–	–	–	–	2000	–
NEX	1	0.75	4	0.5	1	1
Slice thickness/slice gap (mm)	4/0	2.0–2.6/0	4/0.5	4/0.4	4/0	18
Field of view (mm) (adjusted to patient)	320 x 320	320 × 260	400 × 360		380 × 323	480 × 480
Freq/phase matrix	256/256	320/256	128/128		192/128	192/256
Reconstructed voxel size (mm)	0.5 x 0.5 x 4	06 × 0.5 × 1–1.3	–	–	–	–
B value			600–800			–
Approx. scan time (secs)	32	21	64	54	51	50

### Image processing and analysis

**Fig. 1:**
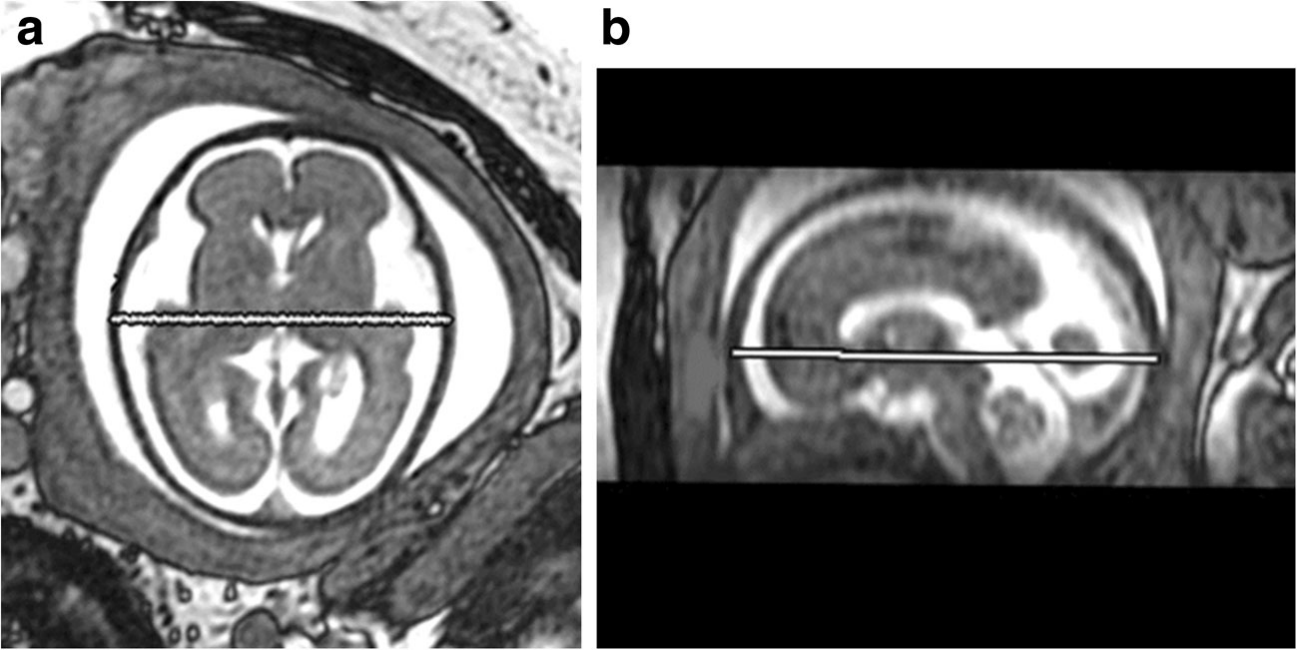
Axial and Sagittal MR images from 3D datasets showing the measurement of bi-parietal diameter (**a**) and occipito-frontal diameter (**b**)

Linear measurements of skull size were made on 2D images reconstructed to the formal orthogonal planes from the 3D datasets by a research MR radiographer (DJ) with over 8 years’ experience of fetal imaging. BPD was measured in the axial plane and OFD in the sagittal plane from the outer table to outer table of the skull (Fig. 1a, b). Head circumference was not measured in this study.

**Fig. 2:**
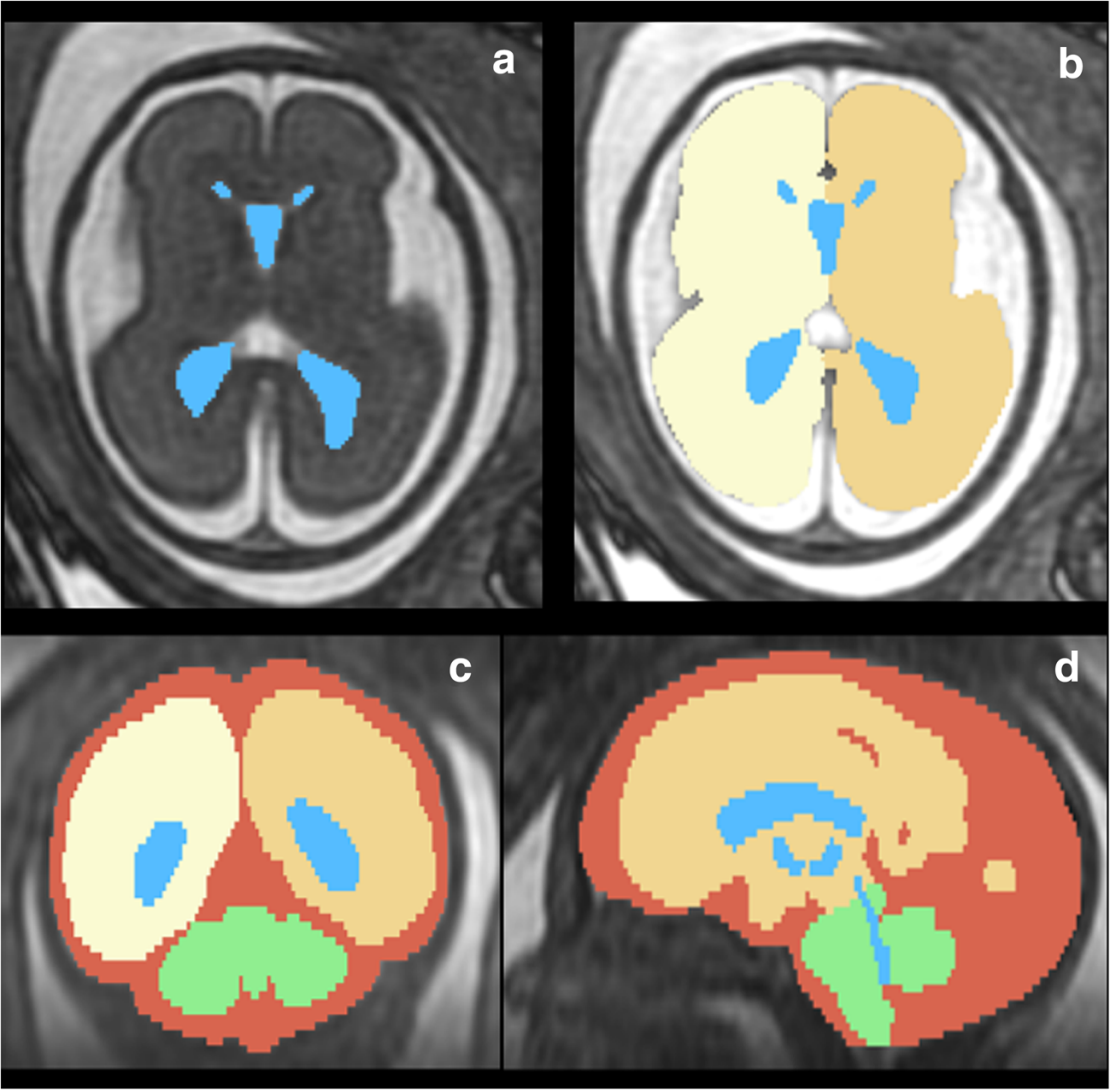
In utero MR imaging of a normal control fetus at 26 gestational weeks showing the regions of interest segmented for volume measurements; (**a**) Axial 3D FIESTA showing the ventricular system segmented in blue and (**b**) cerebral hemispheres represented by cream and yellow. (**c**) Reconstructed coronal and (**d**) sagittal images with the extra-axial spaces shown in red and the cerebellum and brain stem in green

The 3D datasets were analysed further by the experienced research MR radiographer (DJ) and a more junior researcher (CRF) under guidance, using ‘3D Slicer’ software (www.slicer.org [15]). We have previously described good intra- and inter-observer reproducibility of this technique [10, 12] and this has also been confirmed for the current pairing of assessors as part of her training program (data not presented here). The intracranial compartments described below were outlined manually on the axial images (because of higher in-plane resolution) but coronal and sagittal planes were also used to improve accuracy. The ventricular system (including the lateral, third and fourth ventricles, choroid plexus, cerebral aqueduct and cavum septum) was outlined first and the enclosed pixels constitute the VV (Fig. 2a). The brain surface (both cerebral hemispheres, Fig. 2b, brain stem and cerebellum, Fig. 2c, d) was then outlined and the pixels enclosed between that surface and the ventricular system constitute BPV (excluding the VV). The inner surface of the skull was outlined and the pixels enclosed between that construction and the outer surface of the brain constitute EAV (external CSF spaces and the majority of the intracranial vascular compartment) (Fig. 2c, d). The VV, BPV and EAV were summed to estimate the TICV. The volumes of each compartment were visualised as electronic surface models from the model-making algorithm utilised by 3D Slicer. The algorithm also generated the volume data by multiplying the number of voxels by the voxel size belonging to each area segmented. It is important to note that the software used for creating the 3D datasets in this study (3D Slicer) does not have CE-marking and so cannot be used as a clinical tool at present.

### Statistical analysis

The mean and SD were calculated for the measured volumes of BPD, OFD, VV, BPV, EAV and TICV at each gestational age for the control cohort and presented in tabulated form. The volumes for BPD, OFD, VV, BPV, EAV and TICV were also plotted against gestational age and regression analysis used to draw lines representing the mean and the predicted 95% confidence intervals. The best model fit was selected based on the highest adjusted *R*^2^ value and analysis of the residuals.

We compared our normative data for BPD and OFD with published USS data [1] in terms of correlation coefficient and by plotting differences in measurements at each time point. Statistical analysis was performed using SPSS software version 20. It was not possible to make a similar analysis of the intracranial volumes derived from iuMR and USS data because the relevant USS data does not exist.

LMS Chartmaker software, version 2.54 [16], was used to plot smooth curves in order to generate values for the 3rd, 10th, 50th, 90th and 97th centiles according to gestational age based on the original raw data. Values were presented in tabulated form for fetuses 19–36gw only because the 18 and 37gw groups did not have sufficient numbers to calculate reliable standard deviations.

The LMS method [17, 18] normalises the data at each time point by Box-Cox power transformation and summarises the distribution of a measurement by three curves: the median (M), coefficient of variation (S) and skewness, expressed as a Box-Cox power (L). Using penalised likelihood, the curves are fitted by non-linear regression with the extent of smoothing chosen according to the best fit for the data. Following computing of the values for L, M and S, we used the diagnostic tools (worm plots [19] and *Q* tests [20]) within the software to check the goodness of fit of the curves to our data, ensuring accurate centile values.

## Results

### Normative data

**Fig. 3:**
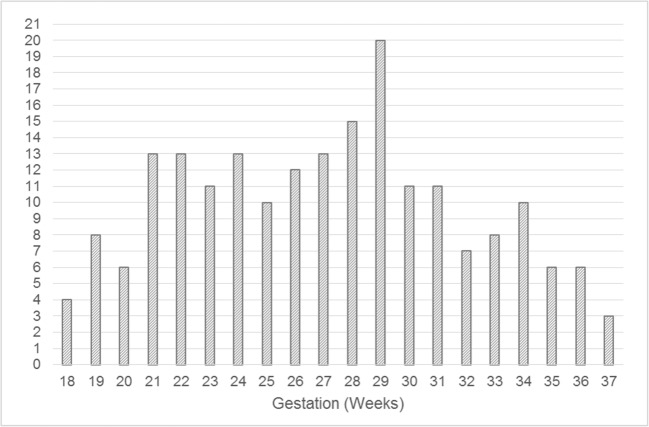
Distribution of 200 normal fetuses reported in this study by gestational age at the time of the in utero MR study

The number of fetuses at each gestational age included in the study are shown in Fig. 3.

The full data sets for BPD, OFD, VV, TBV, EAV and TICV from the control cohort for each gestational age are shown in figures E1a-E1f and in tables E1a-E1f (online). The comparison of BPD and OFD from published USS data and the iuMR presented in this paper is shown in figure E2a-E2d (online).

The tabular summaries of 3rd, 10th, 50th, 90th and 97th centiles from the base data of BPD, OFD, VV, TBV, EAV and TICV for fetuses between 19 and 36gw are shown in Table 2a–f.

**Table 2:** The tabular summaries of 3rd, 10th, 50th, 90th and 97th centiles of bi-parietal diameter (2a), occipito-frontal diameter (2b), ventricular volume (2c), brain parenchymal volume (2d), extra-axial volume (2e) and total intracranial volume (2f)

a. Bi-parietal Diameter (mm)						
Gestation (weeks)	3^rd^	10^th^	50^th^	90^th^	97^th^	SD
19	41.8	43.2	46.1	49.0	50.3	1.4
20	44.8	46.3	49.4	52.5	53.9	2.5
21	47.9	49.4	52.7	56.0	57.5	3.0
22	51.0	52.6	56.0	59.5	61.1	2.4
23	54.1	55.8	59.4	63.0	64.7	2.4
24	57.2	59.0	62.8	66.5	68.3	4.4
25	60.2	62.1	66.0	70.0	71.8	3.3
26	63.2	65.1	69.2	73.3	75.3	3.9
27	66.1	68.1	72.3	76.6	78.6	3.5
28	68.9	70.9	75.3	79.7	81.8	4.8
29	71.6	73.7	78.2	82.7	84.8	3.1
30	74.2	76.4	81.0	85.7	87.8	2.3
31	76.8	79.0	83.8	88.6	90.8	4.6
32	79.3	81.6	86.5	91.3	93.6	4.4
33	81.7	84.1	89.1	94.1	96.4	3.2
34	84.1	86.5	91.6	96.7	99.1	3.3
35	86.5	89.0	94.2	99.3	101.8	5.2
36	88.9	91.4	96.7	101.9	104.4	4.9
b. Occipito-Frontal Diameter (mm)						
Gestation (weeks)	3^rd^	10^th^	50^th^	90^th^	97^th^	SD
19	51.8	53.2	56.3	59.8	61.6	1.5
20	55.5	56.9	60.2	63.9	65.8	3.4
21	59.1	60.6	64.1	68.0	70.0	3.7
22	62.9	64.5	68.2	72.2	74.3	2.6
23	67.0	68.6	72.5	76.7	78.9	3.3
24	71.1	72.9	76.9	81.3	83.5	4.8
25	75.3	77.1	81.3	85.9	88.2	3.1
26	79.3	81.2	85.5	90.3	92.7	2.7
27	83.0	85.0	89.5	94.4	96.9	3.7
28	86.4	88.5	93.1	98.1	100.7	4.4
29	89.6	91.6	96.3	101.5	104.1	2.9
30	92.5	94.6	99.4	104.7	107.3	3.2
31	95.1	97.3	102.2	107.5	110.2	5.9
32	97.6	99.7	104.7	110.1	112.8	4.1
33	99.7	101.9	106.9	112.4	115.2	3.3
34	101.7	103.9	109.0	114.5	117.3	3.8
35	103.6	105.8	110.9	116.5	119.3	6.3
36	105.4	107.6	112.8	118.4	121.3	6.6
c. Ventricular Volume (cm^3^)						
Gestation (weeks)	3^rd^	10^th^	50^th^	90^th^	97^th^	SD
19	1.5	1.9	2.9	4.3	5.2	0.7
20	1.6	2.0	3.1	4.6	5.5	1.4
21	1.7	2.1	3.2	4.8	5.8	0.9
22	1.8	2.2	3.4	5.1	6.1	0.7
23	1.9	2.3	3.6	5.4	6.4	1.1
24	2.0	2.5	3.8	5.7	6.9	1.1
25	2.2	2.7	4.1	6.2	7.4	1.9
26	2.3	2.9	4.5	6.7	8.0	1.5
27	2.5	3.1	4.8	7.2	8.6	2.0
28	2.7	3.3	5.2	7.7	9.3	2.2
29	2.9	3.6	5.5	8.3	9.9	2.2
30	3.0	3.8	5.8	8.7	10.5	1.5
31	3.2	4.0	6.1	9.2	11.1	1.5
32	3.3	4.1	6.4	9.7	11.6	3.1
33	3.5	4.3	6.7	10.1	12.1	2.1
34	3.6	4.5	7.0	10.5	12.6	1.2
35	3.8	4.7	7.2	10.9	13.1	2.9
36	3.9	4.8	7.5	11.3	13.6	2.2
d. Brain Parenchymal Volume (cm^3^)						
Gestation (weeks)	3^rd^	10^th^	50^th^	90^th^	97^th^	SD
19	21.2	23.0	27.5	32.8	35.7	2.2
20	26.5	28.8	34.3	40.9	44.3	7.3
21	32.5	35.3	41.9	49.7	53.8	6.3
22	39.7	43.0	50.9	60.1	65.0	5.6
23	48.4	52.3	61.6	72.5	78.2	6.3
24	58.5	63.0	73.9	86.5	93.2	8.8
25	69.6	74.8	87.3	101.8	109.3	11.8
26	81.5	87.4	101.5	117.8	126.3	11.6
27	94.2	100.8	116.4	134.4	143.7	16.0
28	107.6	114.8	131.9	151.6	161.7	14.9
29	122.1	130.0	148.6	169.8	180.7	13.2
30	138.1	146.6	166.7	189.5	201.2	18.0
31	155.3	164.5	186.1	210.6	223.0	13.7
32	173.4	183.3	206.4	232.4	245.7	26.4
33	192.4	202.9	227.4	254.7	268.6	27.0
34	212.1	223.1	248.7	277.2	291.6	23.0
35	232.2	243.7	270.3	299.6	314.4	18.8
36	252.7	264.5	291.8	321.9	337.0	26.8
e. Extra-axial Volume (cm^3^)						
Gestation (weeks)	3^rd^	10^th^	50^th^	90^th^	97^th^	SD
19	15.7	17.6	22.5	28.7	32.1	5.7
20	19.2	21.5	27.2	34.4	38.4	5.2
21	23.0	25.6	32.2	40.5	45.1	8.3
22	27.3	30.3	37.9	47.3	52.5	4.0
23	32.3	35.8	44.5	55.4	61.3	3.9
24	38.1	42.1	52.3	64.8	71.6	7.3
25	44.6	49.2	61.0	75.4	83.3	7.9
26	51.5	56.9	70.4	87.1	96.2	11.6
27	58.7	64.9	80.2	99.1	109.5	13.0
28	66.1	72.9	90.0	111.1	122.5	20.3
29	73.5	81.0	99.6	122.5	135.0	17.1
30	80.8	88.9	108.8	133.2	146.4	13.6
31	88.0	96.5	117.5	142.9	156.7	13.2
32	95.0	103.8	125.5	151.7	165.8	20.7
33	101.8	110.9	133.1	159.7	173.9	16.3
34	108.6	117.8	140.3	167.0	181.2	23.3
35	115.4	124.7	147.3	174.0	188.0	13.1
36	122.3	131.7	154.3	180.7	194.5	21.2
f. Total Intracranial Volume (cm^3^)						
Gestation (weeks)	3^rd^	10^th^	50^th^	90^th^	97^th^	SD
19	38.9	43.0	52.4	62.9	68.2	6.5
20	48.7	53.6	64.9	77.5	83.8	12.6
21	59.4	65.1	78.3	93.0	100.3	13.7
22	71.7	78.3	93.6	110.5	118.9	8.6
23	86.2	93.8	111.3	130.6	140.3	8.9
24	102.8	111.5	131.5	153.5	164.4	15.5
25	121.5	131.4	153.9	178.6	190.8	18.4
26	142.0	152.9	178.0	205.4	218.9	18.3
27	163.8	175.9	203.4	233.3	248.1	25.5
28	186.7	199.8	229.6	261.8	277.7	20.3
29	210.7	224.8	256.6	290.9	307.8	28.5
30	235.6	250.5	284.2	320.3	338.0	24.9
31	261.3	276.9	312.0	349.7	368.1	23.6
32	287.3	303.5	339.9	378.7	397.6	40.9
33	313.8	330.5	367.7	407.2	426.5	24.7
34	340.7	357.6	395.4	435.4	454.8	23.3
35	368.1	385.1	423.1	463.1	482.5	31.6
36	395.9	412.9	450.7	490.5	509.7	39.0

We also demonstrate in the supplemental material online three fetuses with structural abnormalities in order to show the potential clinical utility of the technique.

## Discussion

We have presented normative data of the skull and intracranial contents from a large cohort (*n* = 200) of control fetuses and individualised data on the intracranial and compartmental volumes between 18 and 37gw. Our approach to measuring intracranial volumes utilises 3D volume MR data with good anatomical resolution and good tissue contrast between CSF and brain. The images are manually segmented, which is time intensive. The recent development of automated methods makes the routine measurement of fetal brain volumes a realistic possibility in clinical practice, enabling the estimation of volumes in shorter time scales with minimal user input [21–24]. However, these methods are guided by templates that require a priori knowledge of normality and have yet to be applied when the normal structure of the brain parenchyma is altered by a pathological process.

Ideally, a study such as this would be supported by comparison with actual, known values but this is not possible when the target is a normal fetus in utero. We were also unable to formally compare our data to that of other MR studies as data is rarely presented in tabulated format [25–27], or the anatomical boundaries for measurements differ [25, 28, 29] alternatively data is limited to a narrow gestational age range [25, 26, 30]. However, estimations of data from the graphs presented by Tilea et al [4], Kyriakopoulou et al [6] and Conte [7] do indicate similar values for BPD and OFD. Review of the results from studies by Kyriakopoulou et al [6] and Gholipour et al [31] also appear to show a good match to our volumetric data.

We were unable to do a within-fetus comparison of USS and MR measurements of BPD or OFD as the time elapse between USS and MR imaging was too long. We have, however, compared our results of BPD and OFD with published results using USS [1] specifically skull measurements made the same way as our method (outer table to outer table of the skull). There was exceptional correlation between the two techniques although there was a tendency for iuMR to measure slightly larger BPD (mean difference 2.36 mm) and smaller OFD (mean difference − 1.49 mm) when compared with USS. We have not been able to compare our intracranial compartmental volume results with data from USS because such data does not exist.

There are anatomical, pathophysiological and neuroimaging advantages to considering the intra-cranial contents as a number of separate compartments distinguished by their contents—brain tissue and CSF. The CSF-containing structures are usefully ascribed to either the ventricular or the extra-axial CSF compartment, both of which are in continuity via the foramina of Magendie and Luschka of the fourth ventricle. The extra-axial CSF compartment, specifically the sub-arachnoid space, also contains some of the larger intracranial vascular structures, although it is usually impossible to differentiate the vascular component from the larger CSF-containing parts on iuMR imaging so they are measured together, unless abnormal. In this paper, we describe three definable and measurable intracranial compartments (VV, BPV and EAV), which, when summated, constitute the TICV.

The TICV and skull dimensions in older children and adults are fixed at any time point because of the rigid nature of the skull after fusion of the fontanelles and cranial sutures. In that situation, the Monroe-Kellie doctrine [32] explains that an increase in volume in one sub-compartment must be accommodated by reductions in the volume of the other sub-compartments or produce raised intracranial pressure. Alternatively, loss of volume from one compartment in an adult must be accompanied by increased volume in one or both of the other compartments or result in reduced intracranial pressure. The situation in the fetus is different because the bones of the calvarium are unfused. This introduces compliance into the system so that increase in volume of intracranial compartment(s) can occur without raising intracranial pressure (within limits). For example, increased pressure and volume of the cerebral ventricles (hydrocephalus) is likely to cause increased TICV and skull dimensions. Using the reverse argument, it is predicted that interference with growth of the fetal brain (a destructive process for example) or reduction in CSF pressure are likely to result in a reduced skull size/TICV when compared to chronologically matched controls.

For these reasons, measurement of skull size is an integral part of the USS assessment of the fetus as any major deviation from normative values on a single study, or a substantial change in the skull size on serial studies, may indicate brain pathology. From the preceding discussion, however, it becomes obvious that that argument is too simplistic because the brain is not the only intracranial structure. A fetus with microcephaly is highly likely to have a small brain, by necessity, but it is not true that a fetus with skull dimensions in the normal range must have a normal sized brain. A disproportionately small brain, in relation to skull size, is known as micrencephaly and often indicates acquired brain pathology. Reduced brain volume is usually accompanied by increased CSF volume, either VV and/or EAV, which maintains the skull dimensions. This distinction is often difficult to make on USS because of poor visualisation of the EAV in particular and is one of the major theoretical advantages of iuMR imaging.

Knowledge of the volumes of the intracranial sub-compartments may assist the differential diagnosis provided by visual assessment of iuMR studies but the methods are still exploratory and formal studies are required to determine the clinical utility of the information provided by volumetric data. The potential of the technique however is shown in the case studies presented in the online supplemental material.

A major strength of this study is the inclusion of 200 fetuses across a wide gestational age range with a minimum of six fetuses for nearly all ages. This has enabled the reliable calculation of centiles for intracranial volumes for gestations 19–36 weeks. Values for 18 and 37 weeks were excluded for the calculation of centiles because we had limited numbers of measurements at these gestations (4 and 3 respectively). The prospective study design and stringent inclusion criteria also allowed a high degree of certainty that the fetuses included were normal. Additionally, fetuses with a family history of abnormalities were also excluded. This is in contrast to other studies whose normative data is derived from fetuses who were either referred for MR imaging due to siblings with abnormalities, had suspected brain abnormality on USS that were subsequently excluded on MR imaging or have a normal brain examination but an abnormality affecting another anatomical area [6, 13, 30]. These cannot be considered a truly normal population, although one study [6] carried out postnatal assessments of the children studied to confirm the data reported was drawn from a normal population. A limitation of this study is that we did not have postnatal imaging or neurodevelopment outcomes for any of the children who had been studied as normal fetuses are not routinely assessed postnatally in the UK. However, previous research has shown that the false positive and false negative rate for detecting abnormalities by prenatal MR is very low [14]. In future studies, we intend to correlate the results with an assessment of outcome. This will also allow comparisons of male and female populations.

A further limitation of our method is the time required for manual segmentation (between 2 and 6 h) which restricts its application routinely in clinical practice. Whilst there has been a great deal of effort to develop automated segmentation methods by several groups [13, 22–26, 28, 33] user input is required to increase precision and there has yet to be a proven clinical utility.

In summary, we have described normative values for a range of cranial and intracranial dimensions in control fetuses between 18 and 37gw. We stress, however, that the software used for creating the 3D datasets (3D Slicer) does not have CE-marking and cannot be used as a clinical tool at present.

## Electronic supplementary material

ESM 1 (DOCX 2.35 mb)

## Abbreviations

2D: Two-dimensional
3D: Three-dimensional
BPD: Bi-parietal diameter
BPV: Brain parenchymal volume
CSF: Cerebrospinal fluid
EAV: Extra-axial volume
gw: Gestational weeks
OFD: Occipito-frontal diameter
TICV: Total intra-cranial volume
USS: Ultrasonography/ultrasound scanning
VV: Ventricular volume
