# Supplementary material for: Normative volume measurements of the fetal intra-cranial compartments using 3D volume in utero MR imaging
Source: Eur Radiol. 2019 Jan 25;29(7):3488–95. doi: 10.1007/s00330-018-5938-5 (PMC6554253; doi:10.1007/s00330-018-5938-5)
Supplement: Supplementary file 1 — (DOCX 2.35 mb) [file 330_2018_5938_MOESM1_ESM.docx]

**Figure E1.** Graphs of mean volume and 95% predicted CI limits for bi-parietal diameter (E1a), occipito-frontal diameter (E1b), ventricular volume (E1c), brain parenchymal volume (E1d), extra-axial volume (E1e) and total intracranial volume (E1f).

**E1a**


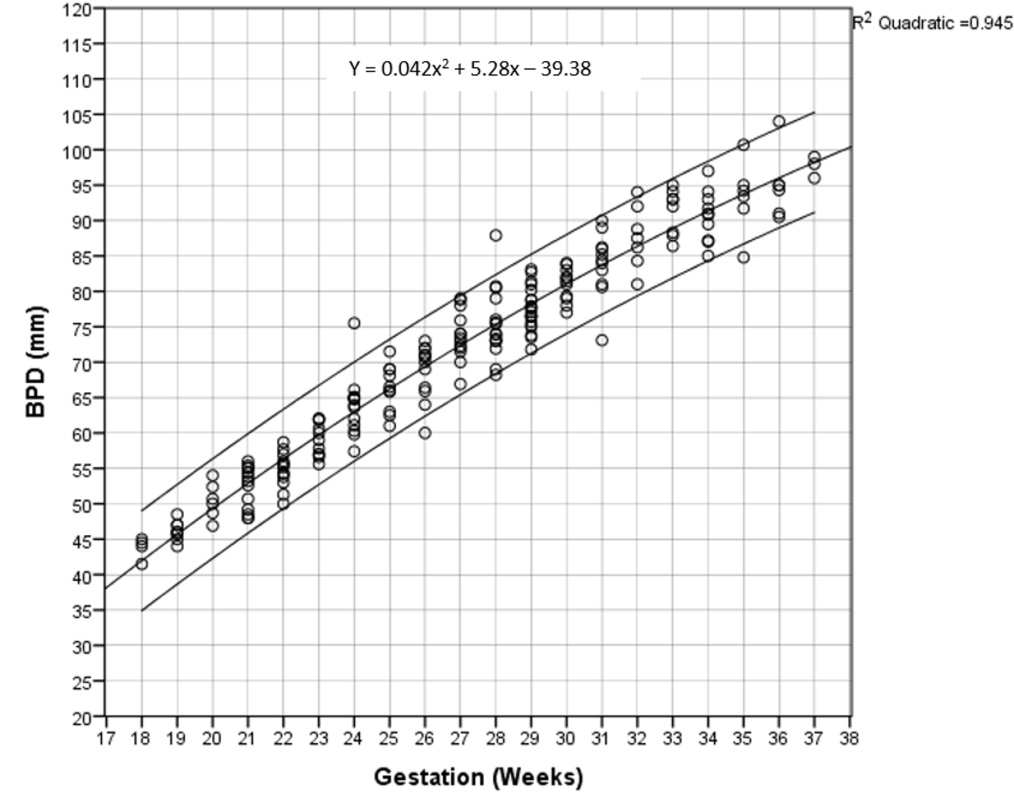


**E1b**


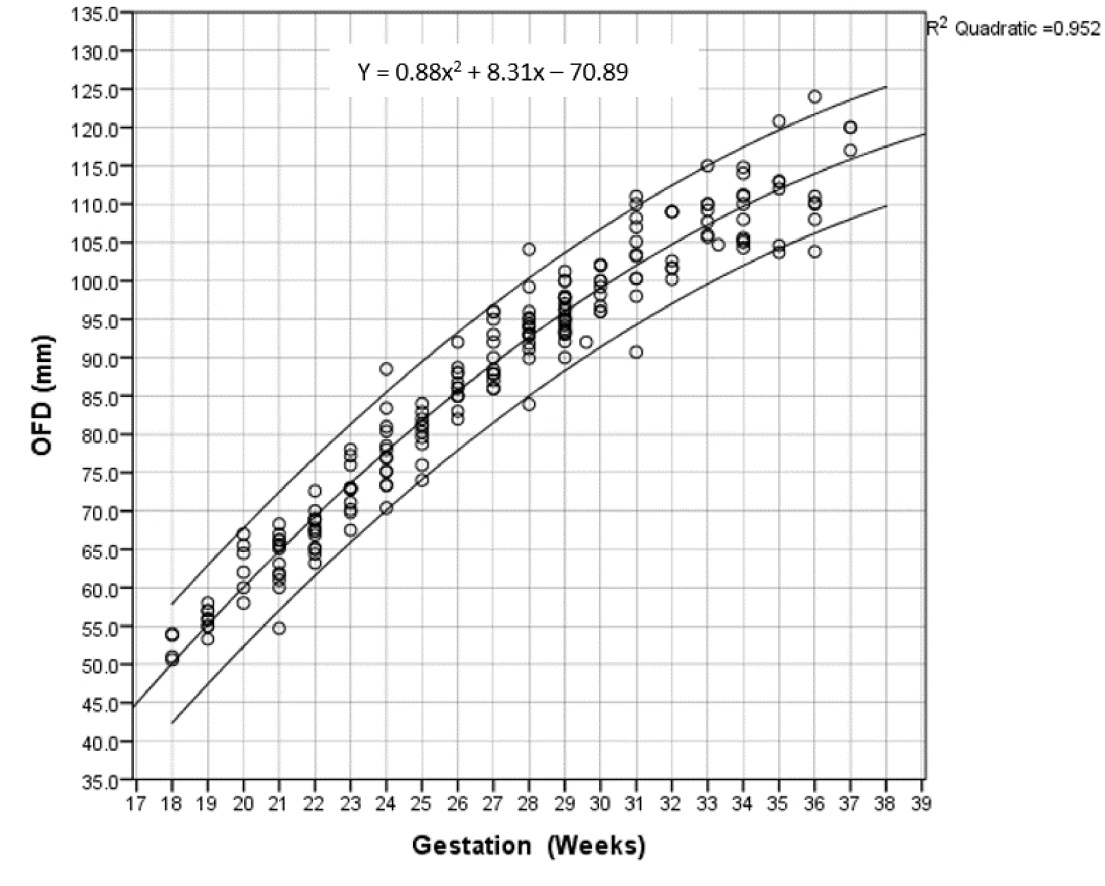


**E1c**


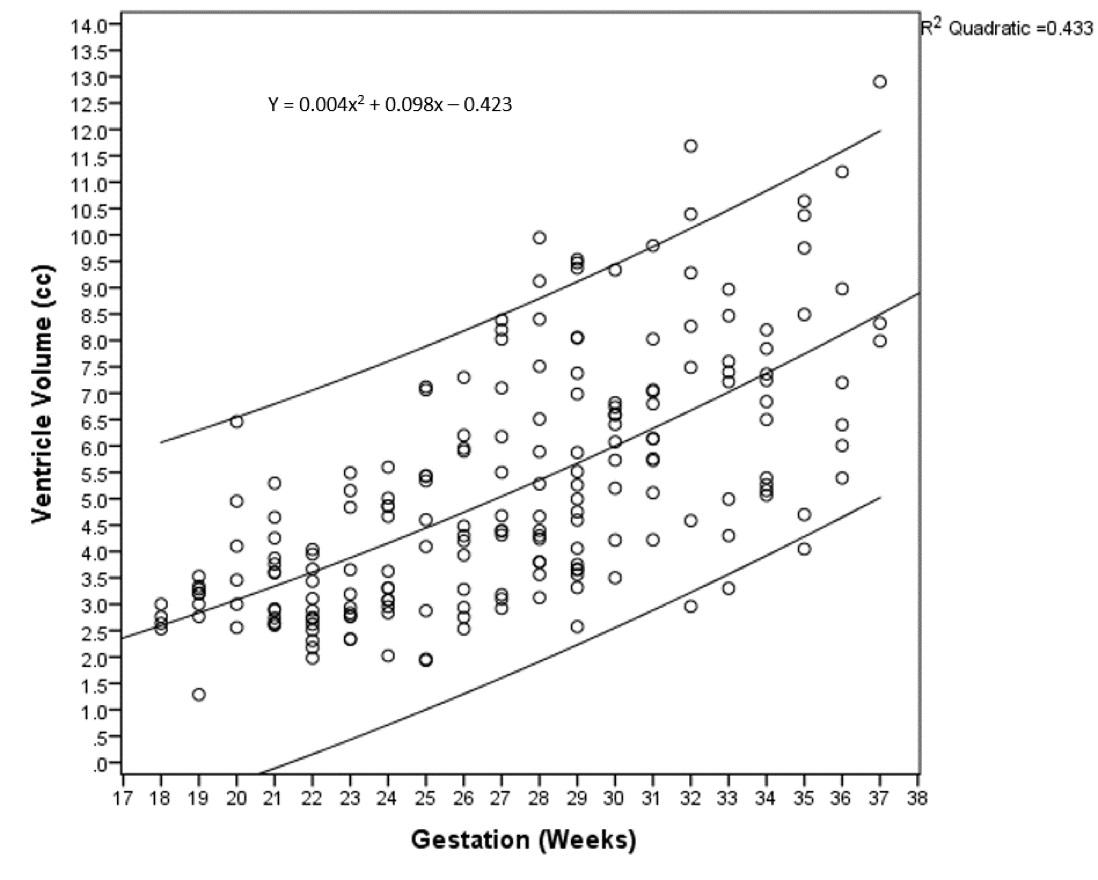


**E1d**

**
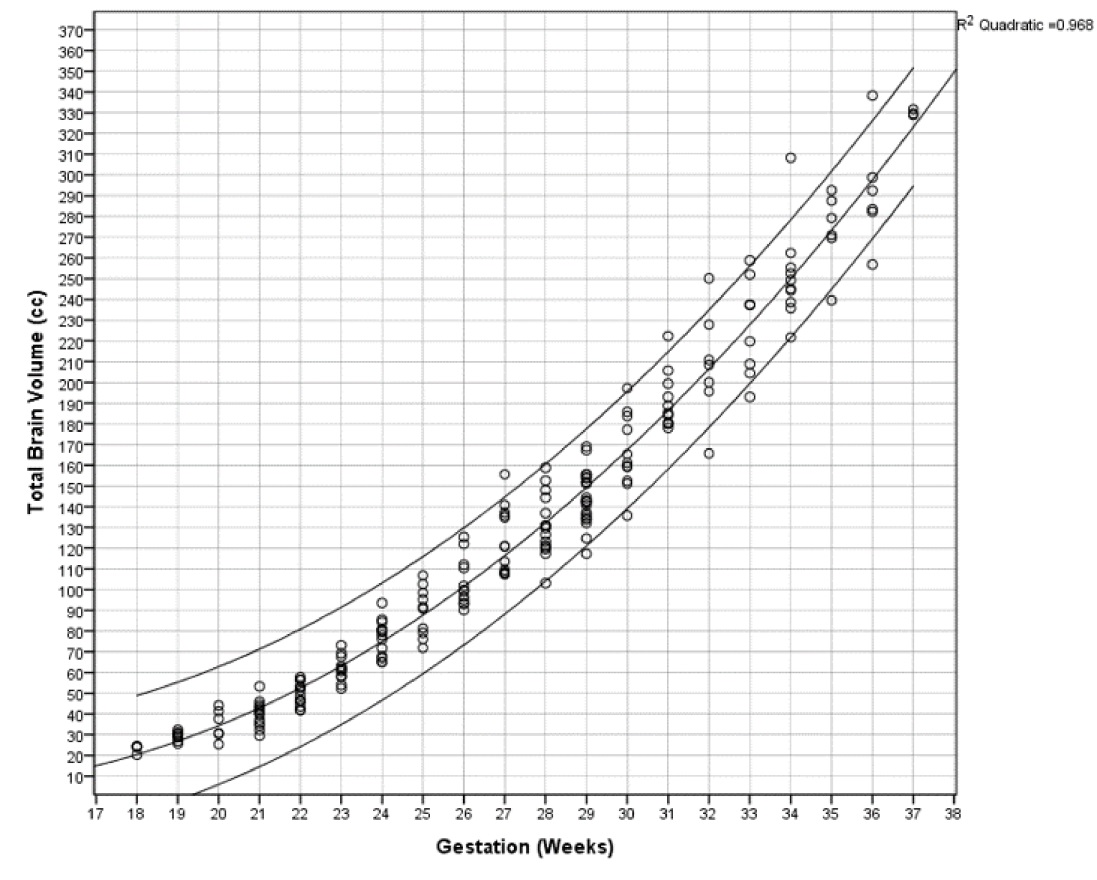
**

**E1e**

**
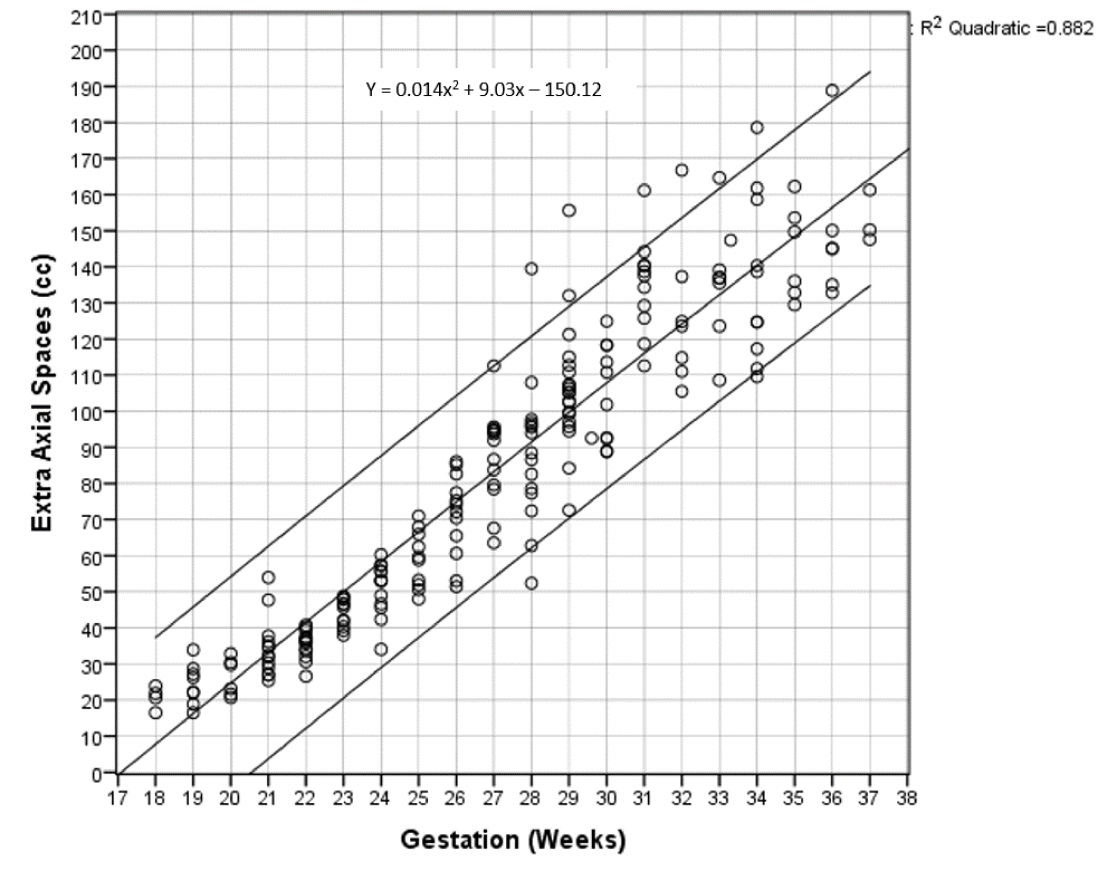
**

**E1f**

**
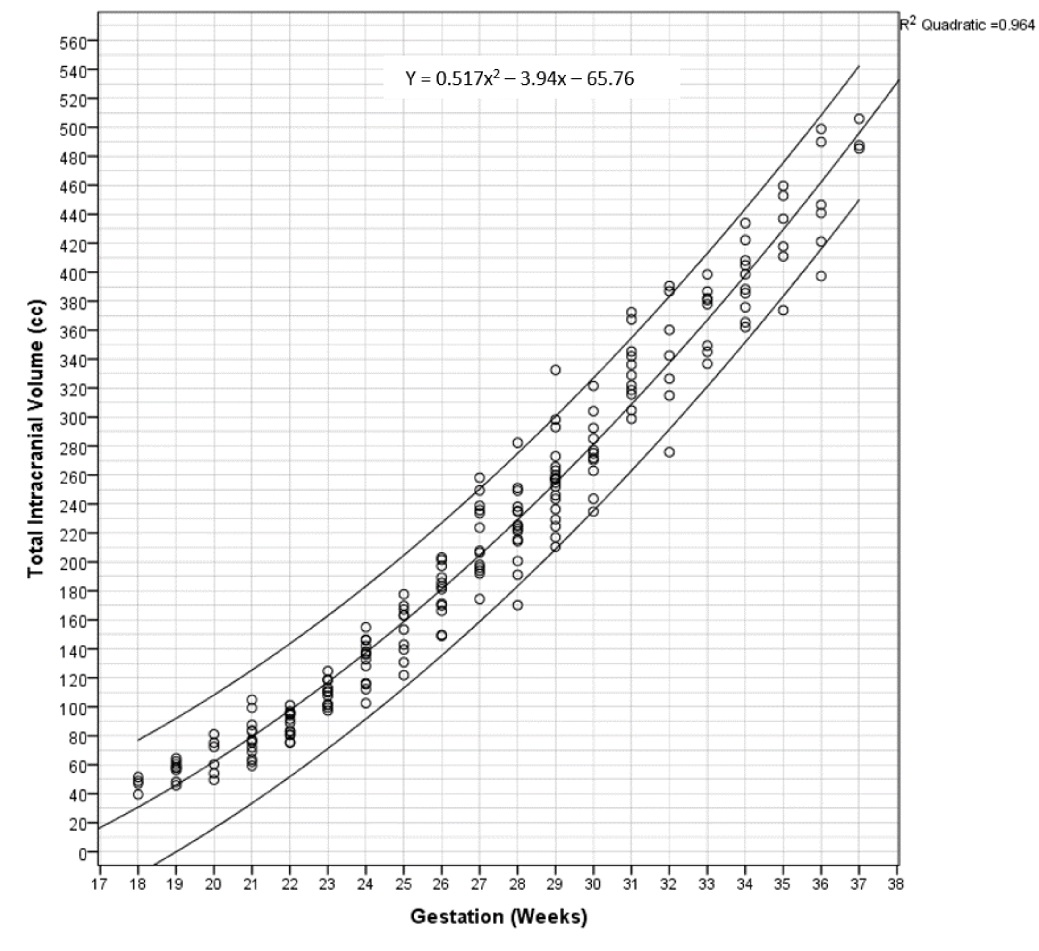
**

**Figure E2.** The comparison of BPD and OFD measured on iuMR in the present study compared with published USS data [1]. The correlation between the two methods at each gestational age is shown for bi-parietal diameter (E2a) and occipito-frontal diameter (E2b) along with graphs showing plots of the differences in mean values between in utero MR and ultrasound by gestational age for bi-parietal diameter (E2c) and occipito-frontal diameter (E2d).

**E2a**

**
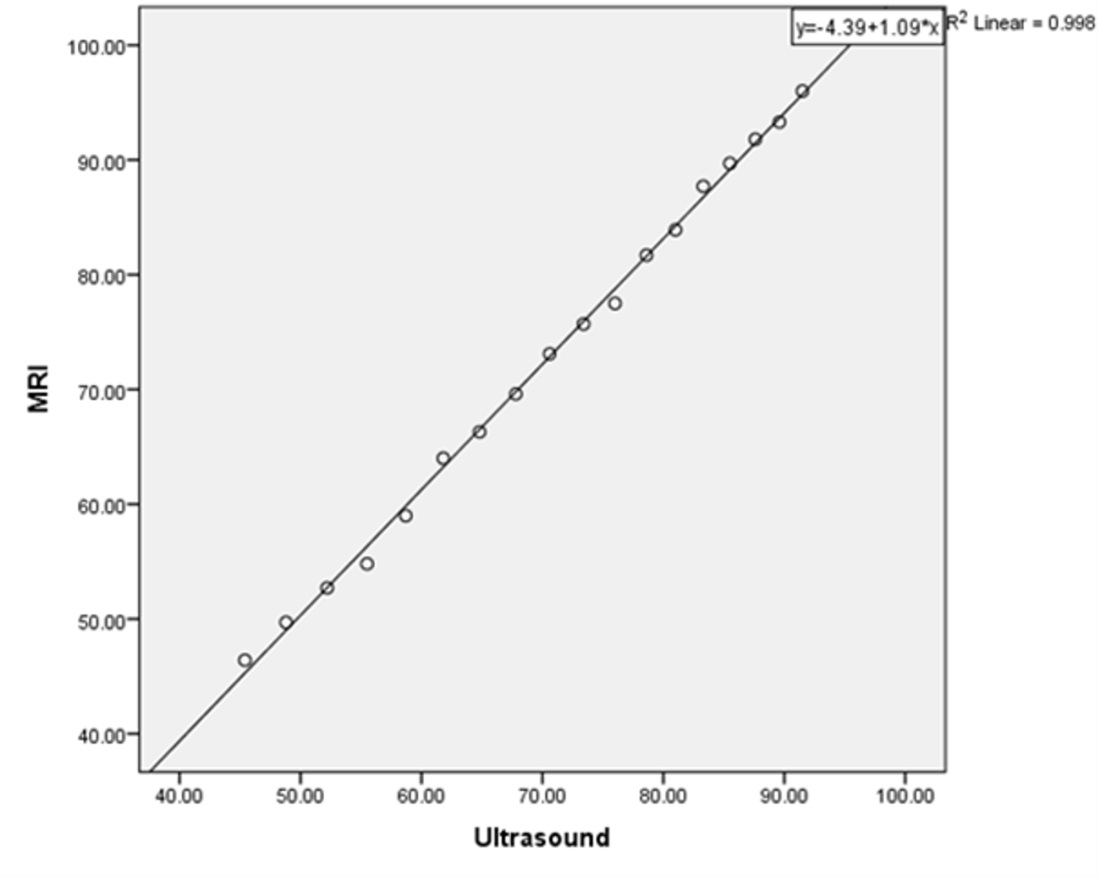
**

**E2b**

**
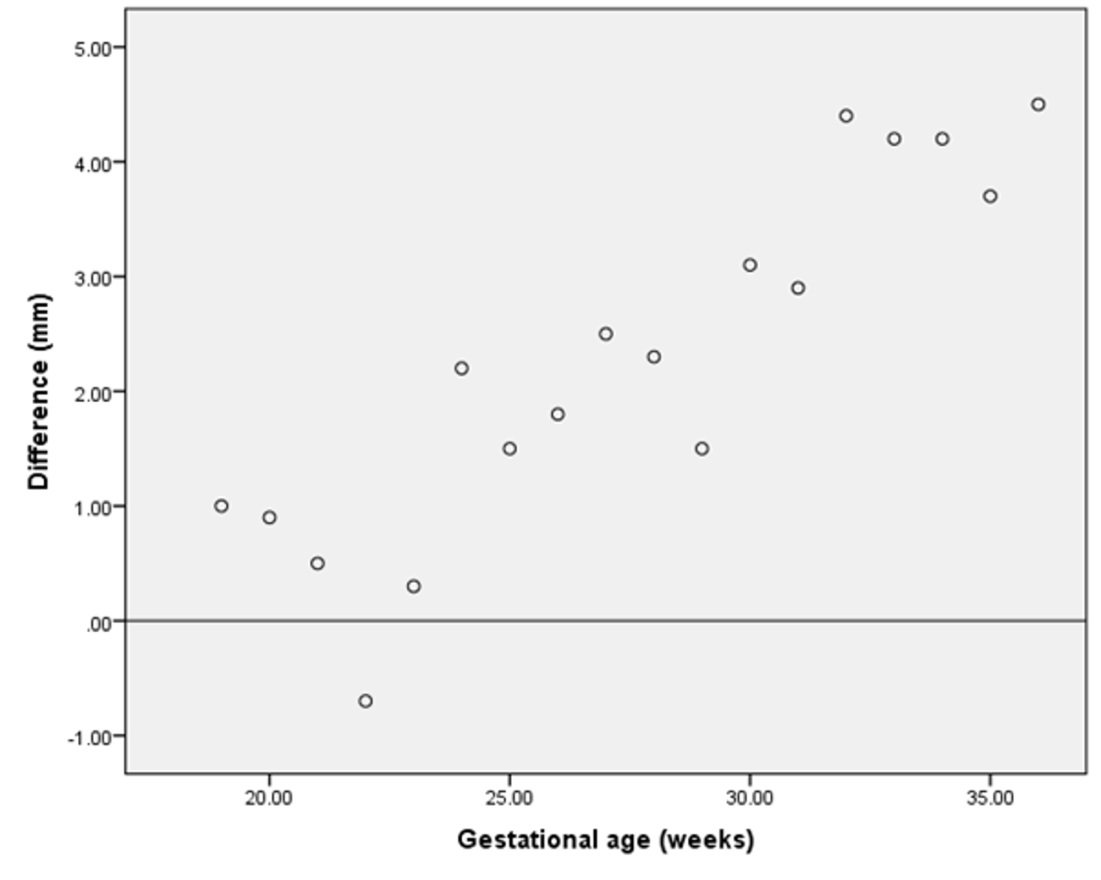
**

**E2c**

**
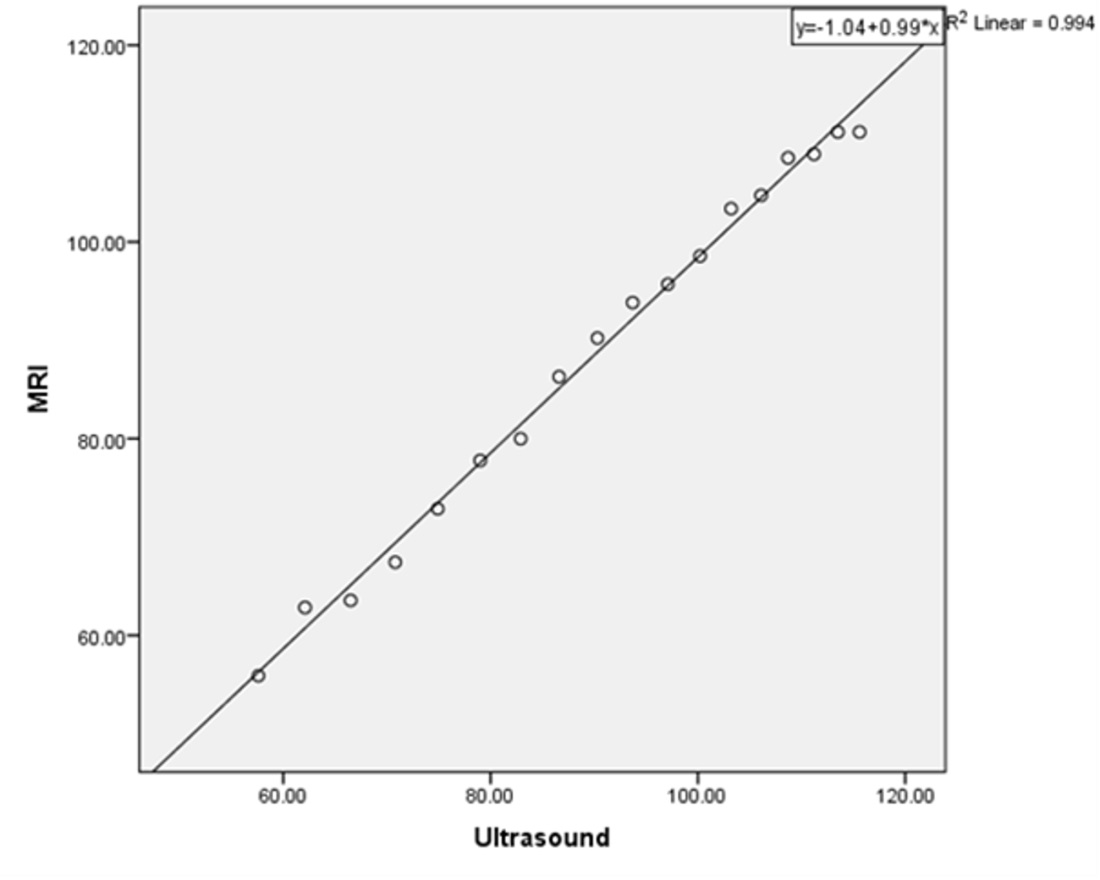
**

**E2d**

**
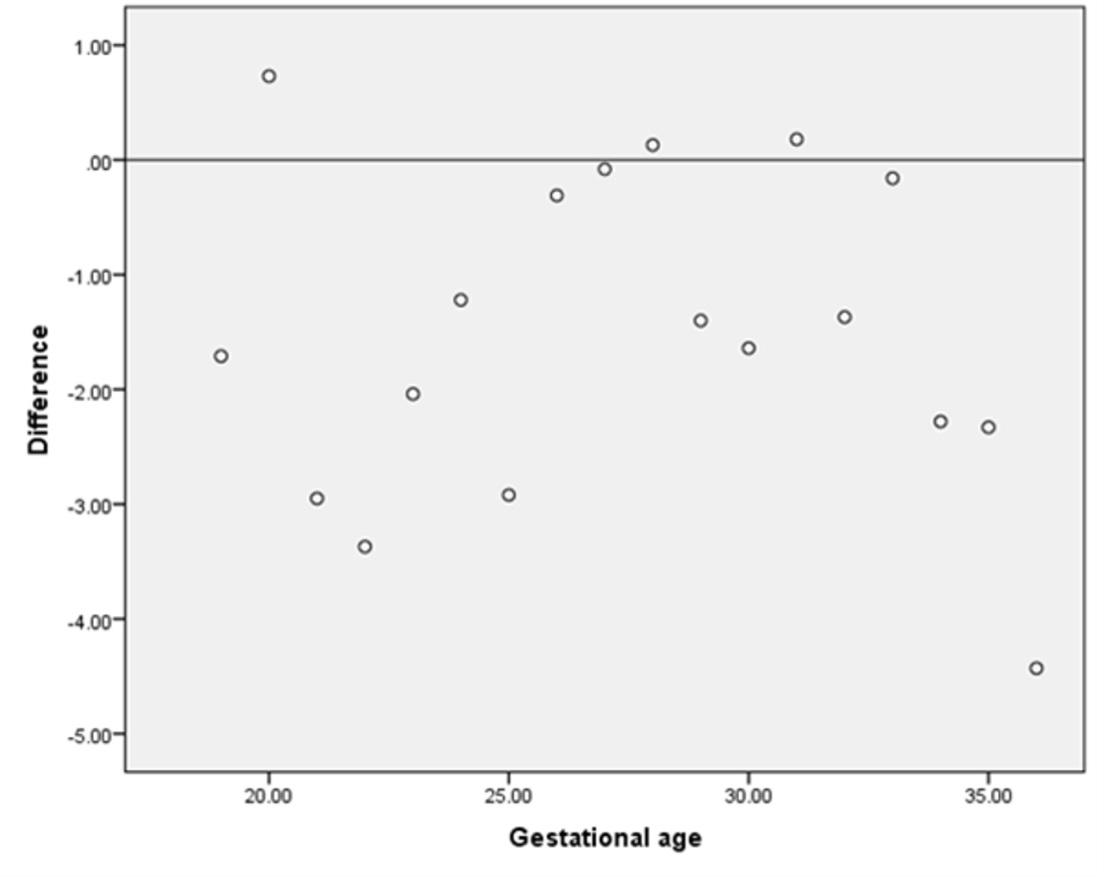
**

**Figure E3.** In utero MR imaging, brain surface models and intracranial volumes of a fetus (23gw) with transplacental infection by cytomegalovirus. See case 1 text for details.

**E3a**

**
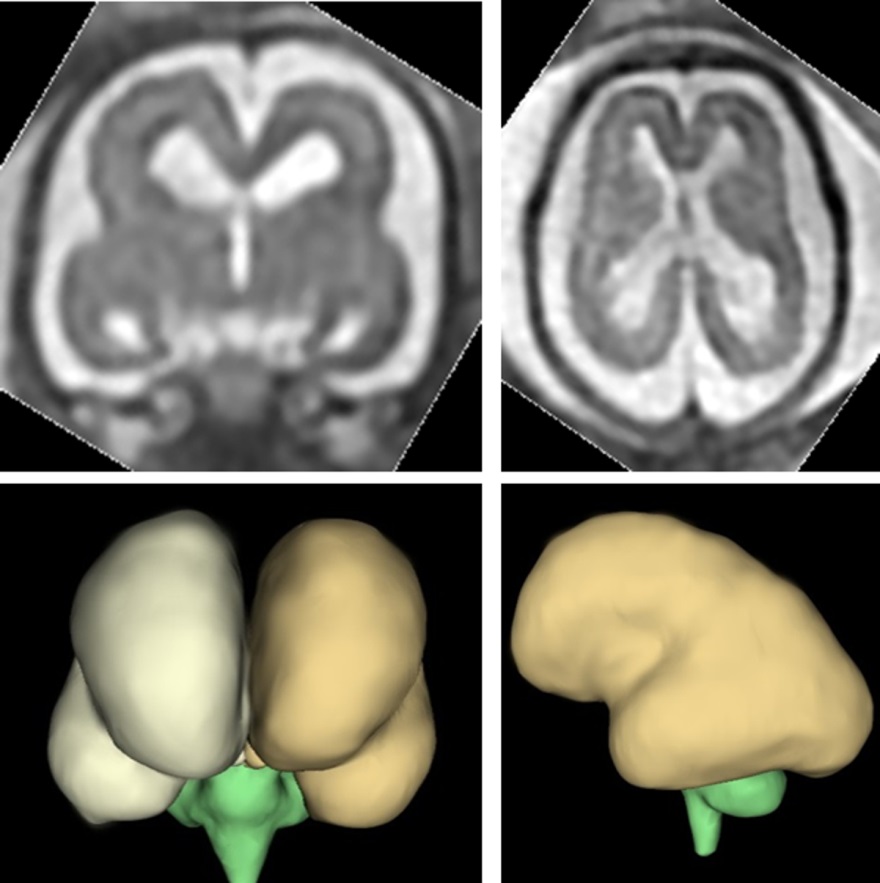
**

**E3b**

**
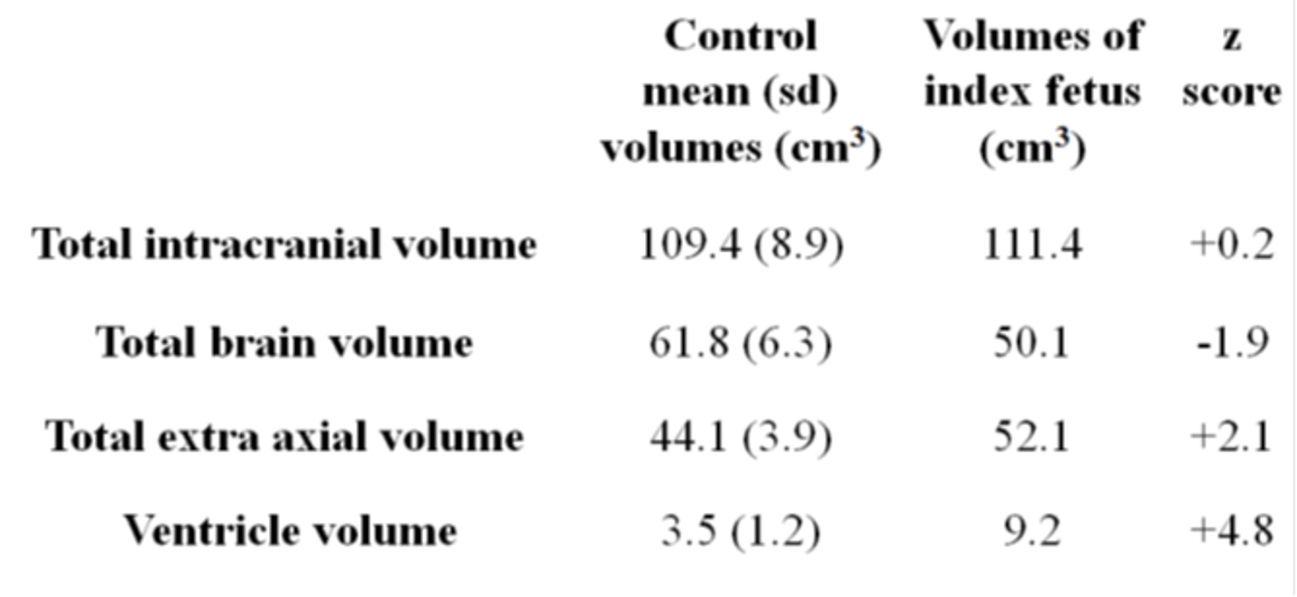
**

**Figure E4**. In utero MR imaging, brain surface models and intracranial volumes in twins, one with hemimegalencephaly (A upper panes) and one with no brain abnormality (B lower panes). See case 2 text for details.

**E4a**

**
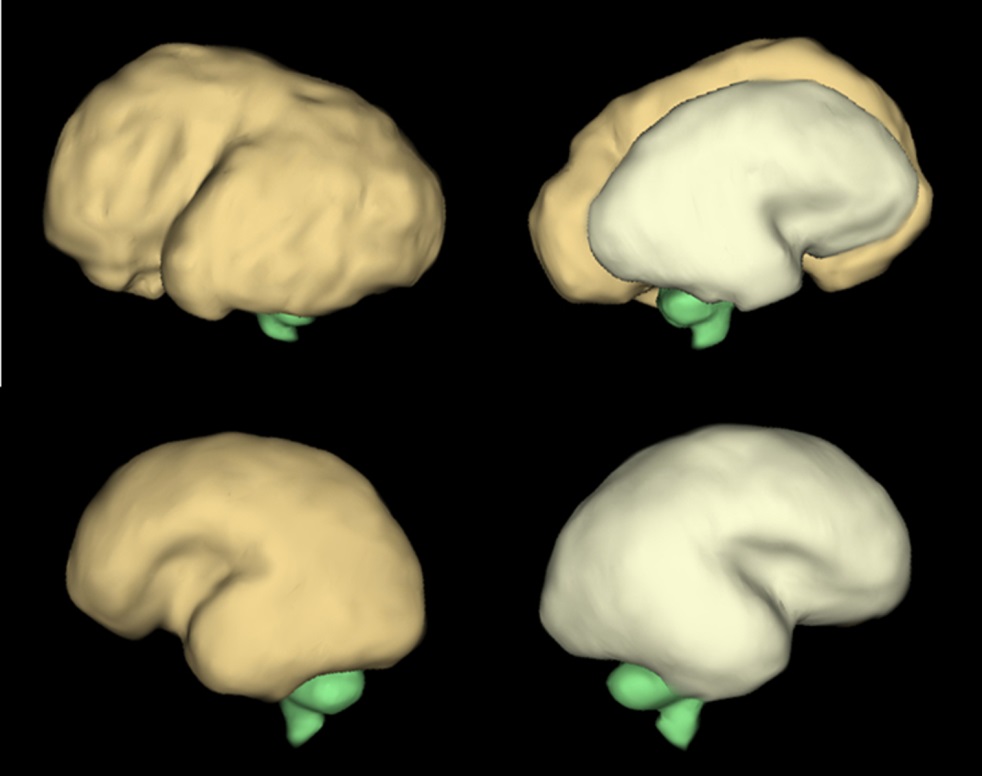
**

**E4b**

**
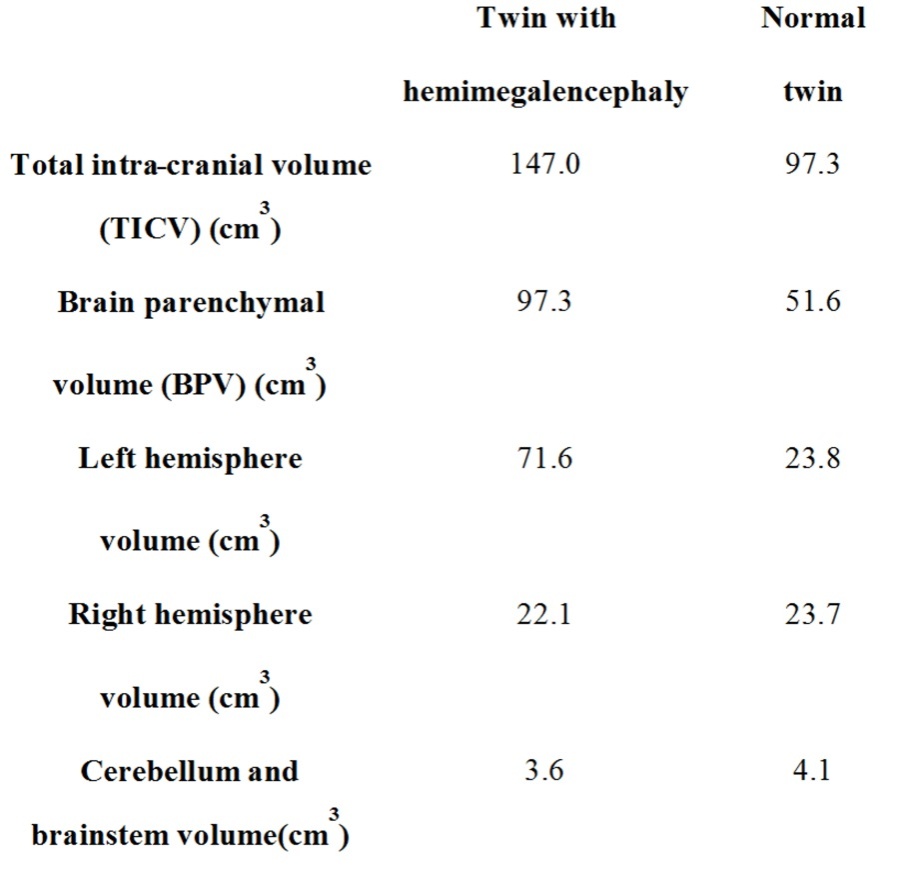
**

**Figure E5**. In utero MR imaging, brain surface models and intracranial volumes of a fetus with ectatic dural sinuses and thrombus. The boundaries of the enlarged dural sinuses have been segmented and included in the surface reconstructions (red). See case 3 text for details.

**E5a**

**
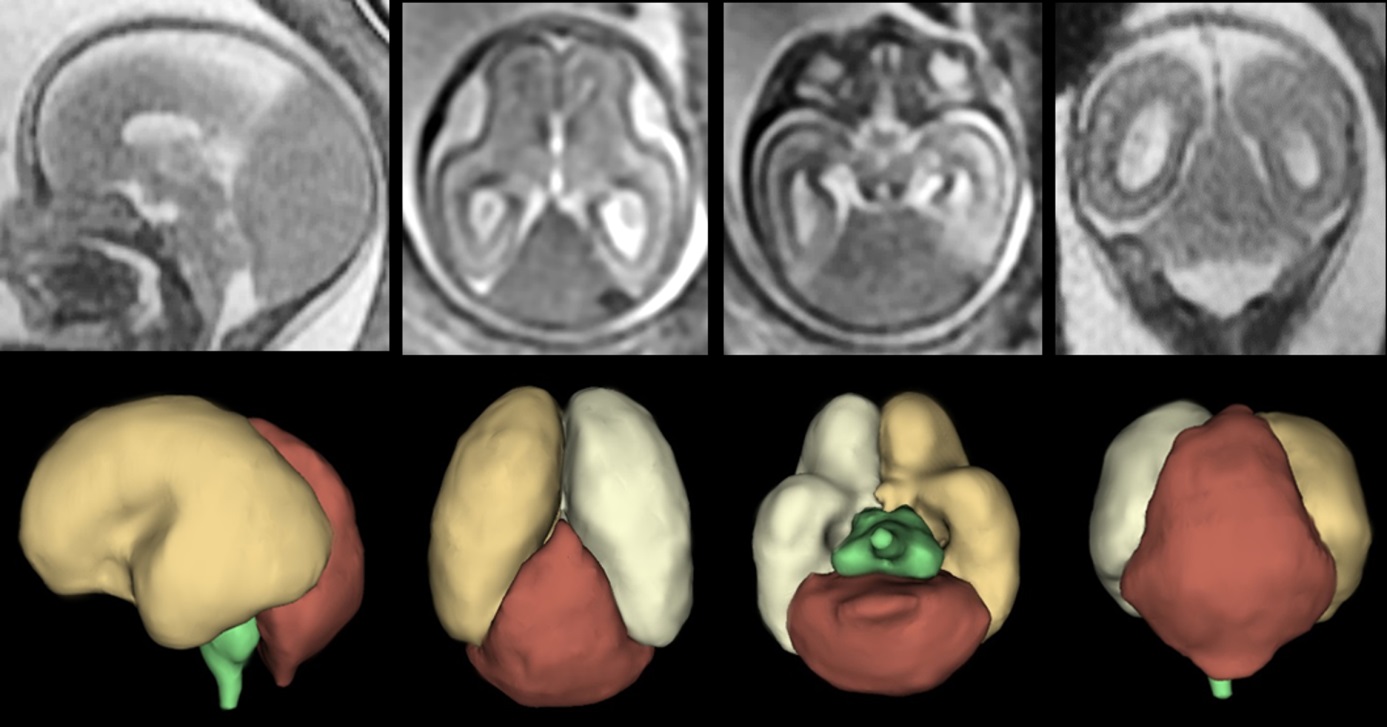
**

**E5b**

**
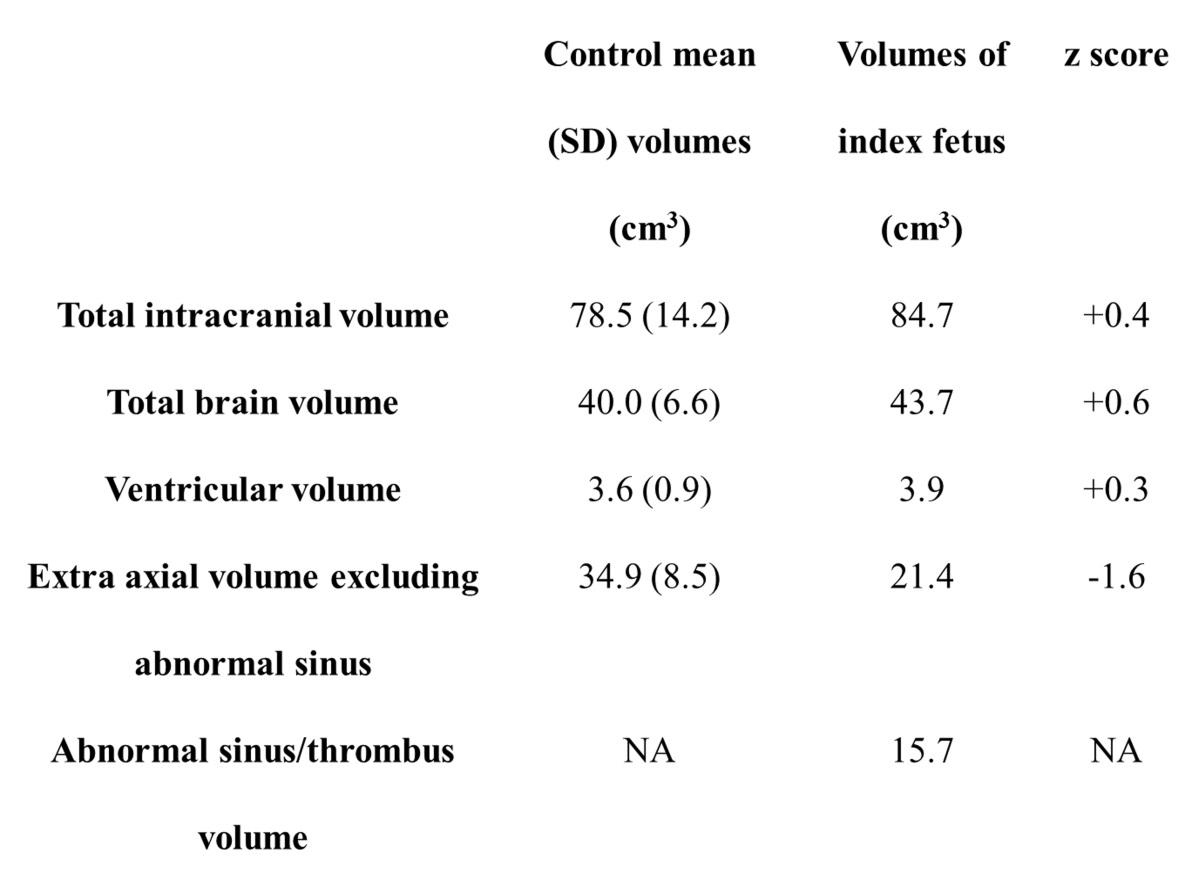
**

**Table E1.** Bi-parietal diameter data


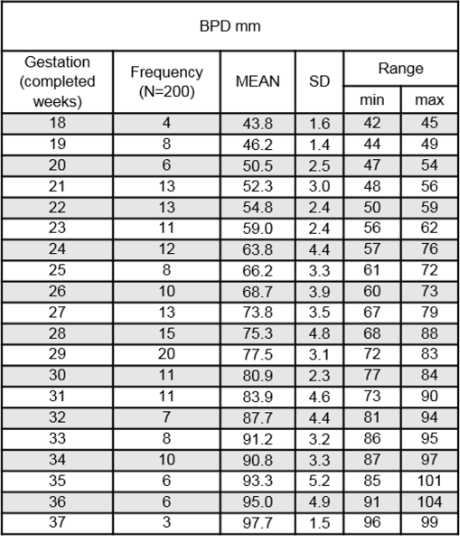


**Table E2.** Occipito-frontal diameter data


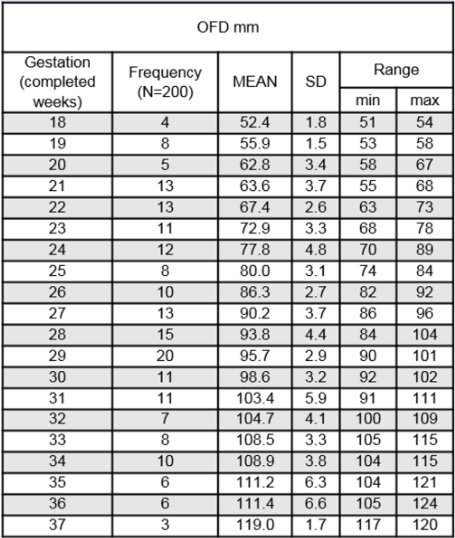


**Table E3.** Ventricular volume data


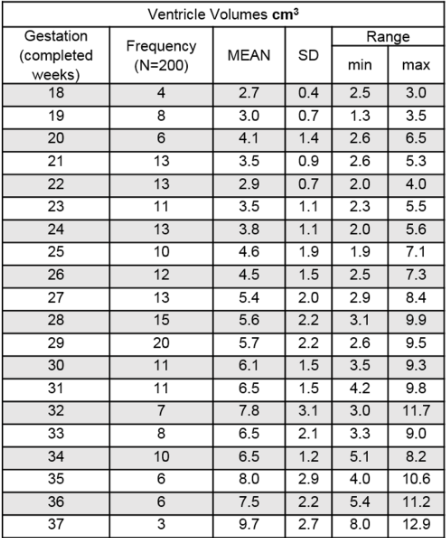


**Table E4.** Brain parenchymal volume data


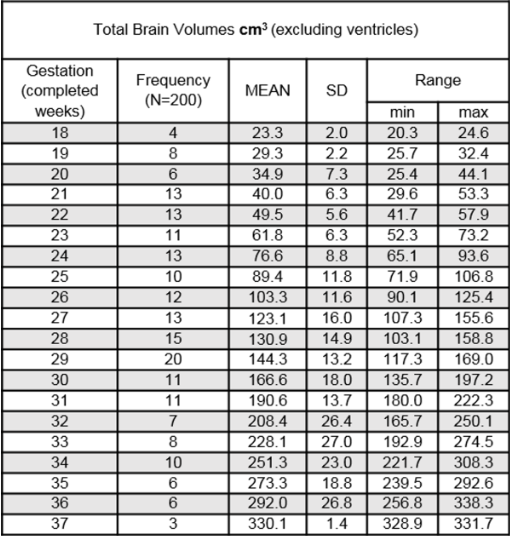


**Table E5.** Extra axial volume data


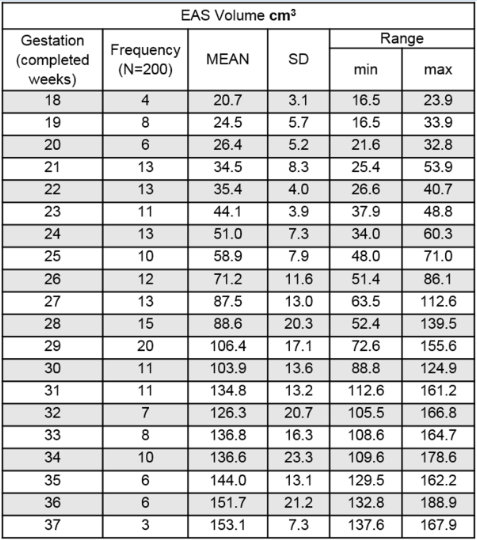


**Table E6.** Total intracranial volume data


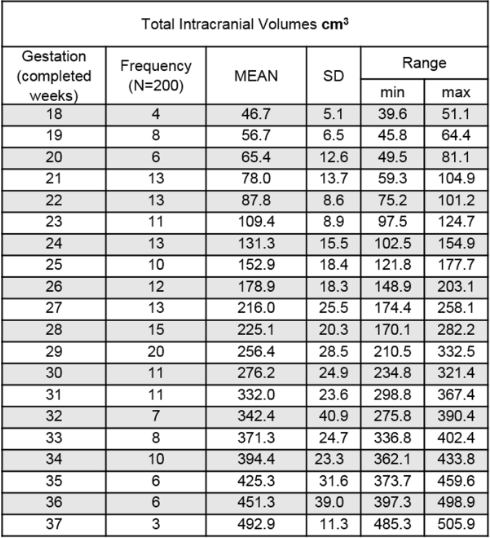


**Clinical cases**

We describe three iuMR studies performed for clinical purposes in order to show the utility of the methods detailed above., It is important to note, however, that the software used for creating the 3D datasets in this study (3D Slicer) does not have CE-marking and so cannot be used as a clinical tool at present. The 3D data analysis presented here was, therefore, not included as part of any clinical reports.

**Clinical cases**

Case 1 A fetus with micrencephaly due to transplacental infection (figure E3)

This fetus had mild ventriculomegaly with normal skull size on USS at 23gw. An iuMR study performed a few days later shows a BPD between 3^rd^-10th centile and an OFD between 10-50^th^ centile. Mild ventriculomegaly is confirmed along with prominence of the extra-axial CSF spaces. In addition, there are indistinct transient layers, an irregular contour to the frontal lobes and poor opercularisation, features consistent with a pervasive cortical formation abnormality. The volumes of the intracranial compartments show normal TICV, reduced TBV (nearly 2sd below the mean) and enlargement of VV and EAV indicating micrencephaly. Ante-natal and postmortem investigations confirmed trans-placental infection by cytomegalovirus, probably arising from re-activation of an earlier infection.

Case 2. A fetus with hemimegalencephaly (figure E4)

A dizygotic twin pregnancy had one fetus with a normal brain on USS at 21gw and an unspecified brain abnormality was recognised in the other. iuMR performed at 22gw confirmed normal appearances and skull dimensions in one twin. The overall skull size of twin 2 was within normal limits (BPD and OFD 50^th^–90^th^ centile) however there was marked asymmetry with a generalized enlargement of the right cranium, cerebral hemisphere and lateral ventricle. The entire right cerebral hemisphere is dysmorphic indicating an extensive cortical formation abnormality consistent with hemimegalencephaly. One of dizygotic twins has a major cortical formation abnormality that effects one cerebral hemisphere only (hemimegalencephaly) and it is possible to use the volume data in order to calculate individual cerebral hemispheres. Demonstrating that the affected hemisphere is large was obvious from the routine imaging but confirming that the contralateral hemisphere has normal volume (in relation to those of the other twin) and appearances is diagnostically valuable. The BPV of both twins are given in figure E4 along with breakdown of the volumes of individual hemispheres, note that the apparently normal sized hemisphere of the twin with hemimegalencephaly has comparable volume to the normal twin.

Case 3. A fetus with ectatic dural sinuses and thrombus (figure E5)

This fetus was thought to have agenesis of the corpus callosum on USS at 20gw and iuMR imaging performed at 21gw excluded that diagnosis but a large extra-axial mass lesion was noted posteriorly. This has the anatomical features and signal characteristics suggestive of thrombus within ectatic duro-venous sinuses [1]. It is not possible to distinguish the subarachnoid space and the vascular elements that make up the EAV structures in normal fetuses but when pathology such as thrombosed sinuses is present it is possible to measure the volume of that part of the vascular compartment. In this fetus the total intracranial volume, total brain volume and ventricular volumes are all close to the mean values as is the total extra-axial volume (CSF + thrombus volume). However, over 20% of the extra-axial volume is occupied by the ectatic dural sinuses and thrombus. The ability to measure an abnormal vascular structure which is separable from the other parts of the EAV is potentially valuable. The pathology demonstrated in this case, ectatic duro-venous sinuses with thrombosis is a rare pathology but the emerging long-term follow up of these cases suggests a relatively benign outcome [2]. Serial measurements of the volume of thrombus may be useful in order to confirm on-going resolution.

References

1. Fanou EM, Reeves MJ, Howe DT et al (2013) In utero magnetic resonance imaging for diagnosis of dural venous sinus ectasia with thrombosis in the fetus. Pediatr Radiol 43:1591–1598

2. Merzoug V, Flunker S, Drissi C et al (2008) Dural sinus malformation (DSM) in foetuses. Diagnostic value of prenatal MRI and follow up. Eur Radiol 18:692–699
